# “Can do, don’t do” are not the lazy ones: a longitudinal study on physical functioning in patients with COPD

**DOI:** 10.1186/s12931-020-1290-9

**Published:** 2020-01-20

**Authors:** Noriane A. Sievi, Thomas Brack, Martin H. Brutsche, Martin Frey, Sarosh Irani, Jörg D. Leuppi, Robert Thurnheer, Malcolm Kohler, Christian F. Clarenbach

**Affiliations:** 1 Pulmonary Division, University Hospital Zurich, Raemistrasse 100, 8091 Zurich, Switzerland; 2 Pulmonary Division, Cantonal Hospital of Glarus, Glarus, Switzerland; 3 Pulmonary Division, Cantonal Hospital of St. Gallen, St. Gallen, Switzerland; 4 Pulmonary Division, Clinic Barmelweid, Erlinsbach, Switzerland; 5 Pulmonary Division, Cantonal Hospital of Aarau, Aarau, Switzerland; 6 University Department of Medicine, Cantonal Hospital Baselland and University of Basel, Basel, Switzerland; 7 Pulmonary Division, Cantonal Hospital of Münsterlingen, Münsterlingen, Switzerland; 8 Zurich Centre for Integrative Human Physiology, University of Zurich, Zurich, Switzerland

**Keywords:** COPD, Physical activity, Exercise capacity, Longitudinal analysis

## Abstract

**Background and objective:**

Reduced physical capacity (PC) and physical activity (PA) are common in COPD patients and associated with poor outcome. However, they represent different aspects of physical functioning and interventions do not affect them in the same manner. To address this, a new PC-PA quadrant concept was recently generated to identify clinical characteristics of sub-groups of physical functioning. The objective of this study was to I) proof the new concept and to verify their differentiating clinical characteristics, II) evaluate the consistency of the concept over time, III) assess whether patients changed their quadrant affiliation over time, IV) and to test if changes in quadrant affiliations are associated with changes in clinical characteristics.

**Methods:**

In a longitudinal, prospective, non-interventional cohort with mild to very severe COPD patients, PC and PA as well as respiratory variables, COPD-specific health status, comorbidities, survival, and exacerbations were yearly assessed.

**Results:**

Data from 283 patients were analysed at baseline. Mean (min/max) follow-up time was 2.4 (0.5/6.8) years. The PC-PA quadrants could be characterized as follows: I) “can’t do, don’t do”: most severe and symptomatic, several comorbidities II) “can do, don’t do”: severe but less symptomatic, several comorbidities III) “can’t do, do do”: few patients, severe and symptomatic, less comorbidities IV) “can do, do do”: mildest and less symptomatic, less comorbidities, lowest exacerbation frequency. Of the 172 patients with at least one follow-up, 58% patients never changed their quadrant affiliation, while 17% declined either PC, PA or both, 11% improved their PC, PA or both, and 14% showed improvement and decline in PC, PA or both during study period. None of the clinical characteristics or their annual changes showed consistent significant and relevant differences between all individual sub-groups.

**Conclusion:**

Our findings suggest that there are no clinical characteristics allowing to distinguish between the PC-PA quadrants and the concept seems not able to illustrate disease process. However, the already low PA but preserved PC in the “can do, don’t do” quadrant raises the question if regularly assessment of PA in clinical practice would be more sensitive to detect progressive deterioration of COPD compared to the commonly used PC.

**Clinical trial registration:**

www.ClinicalTrials.gov, NCT01527773.

## Introduction

Chronic obstructive pulmonary disease (COPD) is frequently accompanied by impaired physical capacity (PC) and reduced daily physical activity (PA), both arising in early disease stages [1]. Moreover, COPD patients are less physically active than patients with other chronic diseases, such as diabetes and rheumatoid arthritis [2]. Both, reduced ability to perform PA and PC, are known to be associated with a poor outcome (impaired health status, increased healthcare utilization) and are strong predictors for all-cause mortality in COPD [3, 4]. PC and PA represent two different aspects of physical functioning. While PC represents the ability to perform activity (a set of attributes), PA represents what people really do during their daily routine (a complex behaviour) [5]. A recent longitudinal assessment of the courses of PC and PA in COPD patients showed that PC remained stable despite a substantial annual decrease in PA [6]. This finding leads to our hypothesis, that the longitudinal decline in PA cannot be explained by a concomitant reduction in exercise tolerance. Furthermore, pulmonary rehabilitation shows a positive impact on PC [7] but incongruent findings on PA improvement [8], suggesting that an improvement in PC does not consistently lead to an increase in PA. Further interventions such as PA counselling or long-term oxygen therapy showed variable effects on PA enhancement [9], but studies comparing the effects of these interventions on PC and PA are missing. This implies that interventions may have to be individually tailored according to patients’ impairments in PC or PA, or both. According to this new approach, Koolen et al. [10] recently developed a PC-PA quadrant concept with PC (“can do”) and PA (“do do”) plotted against axes. This PC-PA quadrant concept identifies sub-groups of physical functioning and comparison of different clinical characteristics may provide an explanation for the discrepancies between PA and PC in individual COPD patients.

To address the request of the authors to proof their newly introduced PC-PA quadrant concept in another heterogeneous COPD cohort [10], we performed the analysis accordingly and verified their findings regarding differences in clinical characteristics in our cohort. Furthermore, additional characteristics were compared among the quadrants. Due to our longitudinal study design, we assessed I) the consistency of the concept over time, II) whether patients changed their quadrant affiliation over time, III) and if changes in quadrant affiliations are associated with changes in clinical characteristics.

## Methods

### Subjects

In the prospective, non-interventional cohort project “The Obstructive Pulmonary Disease Outcomes Cohort Study (TOPDOCS)” patients with already diagnosed mild to very severe COPD from seven pulmonary outpatient clinics in Switzerland were included. Study period was from October 2010 to December 2017 and took place during outpatient visits or hospital stays. Patients were scheduled for initially three annual study visits (some patients extended their participation up to seven study visits). This analysis included data from 283 COPD patients out of the TOPDOCS cohort. Patients aged between 40 and 75 years at inclusion with confirmed COPD according to Global Initiative for Chronic Obstructive Lung Disease (GOLD)-guidelines [11] were assessed for eligibility. Patients were excluded if they suffered from mental or physical disability precluding informed consent or compliance with the protocol. In case of a COPD exacerbation, patients were included into the study or called up for follow-up visits with a delay of at least 6 weeks.

The study was conducted in accordance with the declaration of Helsinki and all subjects gave written informed consent to participate. The Ethics Committee of the Canton of Zurich approved the study (EK-ZH-NR: 1734 and 2011–0106) and the study is registered at www.ClinicalTrials.gov, NCT01527773.

### Measurements

#### Physical capacity

The 6-min walking distance (6MWD) was annually assessed according to the American Thoracic Society (ATS) guidelines [12]. The 6-min walking test (6MWT) was performed on a 75 m indoor track, and patients were told to walk as far as possible within six minutes. Oxygen supplementation was allowed if required. At start and end of the test, peripheral oxygen saturation was measured by oximeter (PC-60C Fingertip Oximeter, Shanghai International Trading Corp. GmbH, Hamburg, Germany). Percentage of predicted values of 6MWD was calculated by reference equation of Enright et al. [13] The minimal important difference (MID) for 6MWD in COPD patients is estimated as 25 m [14].

#### Physical activity

The number of steps per day was measured by a validated, triaxial accelerometer of a multisensory activity monitor (SenseWear Pro™; Bodymedia Inc., Pittsburgh, PA, USA) without a display (patient were blinded to the amount of steps per day) [15]. The monitor was worn on the upper left arm for 7 consecutive days once a year. The threshold for valid data from the armband was set at 4 days with a minimum of 22.5 h/day. Seasonality was considered in the analysis. A change of 600 to 1100 steps per day is supported to be the MID in COPD patients [16].

#### PC-PA quadrant concept

As described in the manuscript by Koolen and colleagues [10], the PC-PA quadrants were plotted as follows: “can’t do, don’t do” quadrant with low PC (6MWD < 70% pred.) and low PA (number of steps per day < 5000 steps); “can do, don’t do” quadrant with preserved PC (6MWD ≥70% pred.) but low PA (number of steps per day < 5000 steps); “can’t do, do do” quadrant with low PC (6MWD < 70% pred.) but preserved PA (number of steps per day ≥5000 steps); “can do, do do” quadrant with preserved PC (6MWD ≥70% pred.) and preserved PA (number of steps per day ≥5000 steps).

To assess the longitudinal course of the PC-PA quadrant concept, patients were categorized into four “changing groups” according to their quadrant affiliations over time. COPD patients who never changed their PC-PA quadrant during study period were categorized as “remainer”, “decliner” were patients who decreased their physical functioning (decline in 6MWD < 70% pred. and/or number of steps per day < 5000 steps) at least once, “improver” increased their physical functioning over time (increase in 6MWD ≥70% pred. and/or number of steps per day > 5000 steps) at least once, and “waverer” included patients who increased and decreased their physical functioning over time (increase and/or decrease in 6MWD and/or increase and/or decrease in steps per day) at least once.

#### Respiratory variables

Standard pulmonary functional testing was performed according to ATS/ERS guidelines [17, 18] to measure forced expiratory volume in one second (FEV_1_), residual volume to total lung capacity (RV/TLC) ratio, and diffusing capacity of the lung for carbon monoxide (DLco). Only values after bronchodilation were reported. Disease severity was assessed by spirometric GOLD stages (stage 1–4) and COPD risk groups (risk score A-D) [19].

#### Blood gas analysis

Daytime arterial blood gas analysis was performed to assess partial pressure of oxygen (PaO_2_), of carbon dioxide (PaCO_2_) and oxygen saturation (SaO_2_) after 5 min of rest (ABL 700 series blood gas analyzer, Radiometer, Copenhagen). Measurement was performed native, except few patients with consistent oxygen supplementation during blood gas analysis over all study visits.

#### COPD-specific health status

Severity of dyspnea was assessed by modified medical research council (mMRC) scale [20], for which an MID is not available due to its poor evaluative properties to detect changes in dyspnea [21]. COPD Assessment Test (CAT) was performed to measure the impact of COPD symptoms on health status [22] with an estimated MID of 2 points [23].

To assess states of anxiety and depression, the self-administered Hospital Anxiety and Depression Scale (HADS) was used. The questionnaire is composed of two 7-item sub-scales (HADS-A for anxiety and HADS-D for depression), sub-scores ranging from 0 to 21 with higher scores indicating more severe distress. A score of 0 to 7 is suggested to be non-cases, 8 to 10 as possible cases, and > 10 as probable cases of clinical anxiety or depression, respectively [24]. A change of 1.5 points is suggested to be the MID [25].

#### Comorbidities and survival

Comorbidities were annually assessed by review of the documented medical history, conducting clinical interviews and clinical examinations. To classify comorbidities, the International Classification of Diseases-Tenth Revision [26] was used. The number of comorbidities was calculated by sum up the various diseases.

Last update in April 2019 was used to evaluate patients who died within or after the study period and to assess survival time. Survival time was defined as time from baseline visit to death or April 2019 in patients who stayed alive. In patients who were lost of follow-up, survival time was not calculated.

#### Exacerbation history

An acute exacerbation (AE) was defined as an increase in patient’s dyspnea, cough and/or sputum with prescription of antibiotics and/or corticosteroids. Severe exacerbation was determined as hospital admission due to AE. Annual acquisition of number of AEs during the preceding year was performed and patients were categorized into infrequent exacerbators (0–1 AE per year) and frequent exacerbators (≥2 AEs per year) [27]. To get the most accurate information on AE, patients reports were compared with documents from the general practitioner, pulmonologist and hospital.

### Data analysis and statistics

All results are shown as mean values (standard deviation (SD)) or median (25%/75% quartiles) unless otherwise stated. Statistical analysis was performed with STATA 15.1 (StataCorp, Texas, USA).

Overall differences in PC-PA quadrants and in changing groups were compared by Kruskal-Wallis rank sum test, due to the small sample size in some of the sub-groups, and Chi square tests. For continuous variables, non-parametric post-hoc tests were used to compare the sub-groups among each other, *p*-values were Bonferroni corrected. Post-hoc analysis of categorical variables were assessed by Chi square test. For longitudinal data analysis, median annual change in each patients’ clinical characteristics was calculated.

A two-sided p-value of < 0.05 was considered to be statistically significant.

## Results

### Study participants

**Fig. 1:**
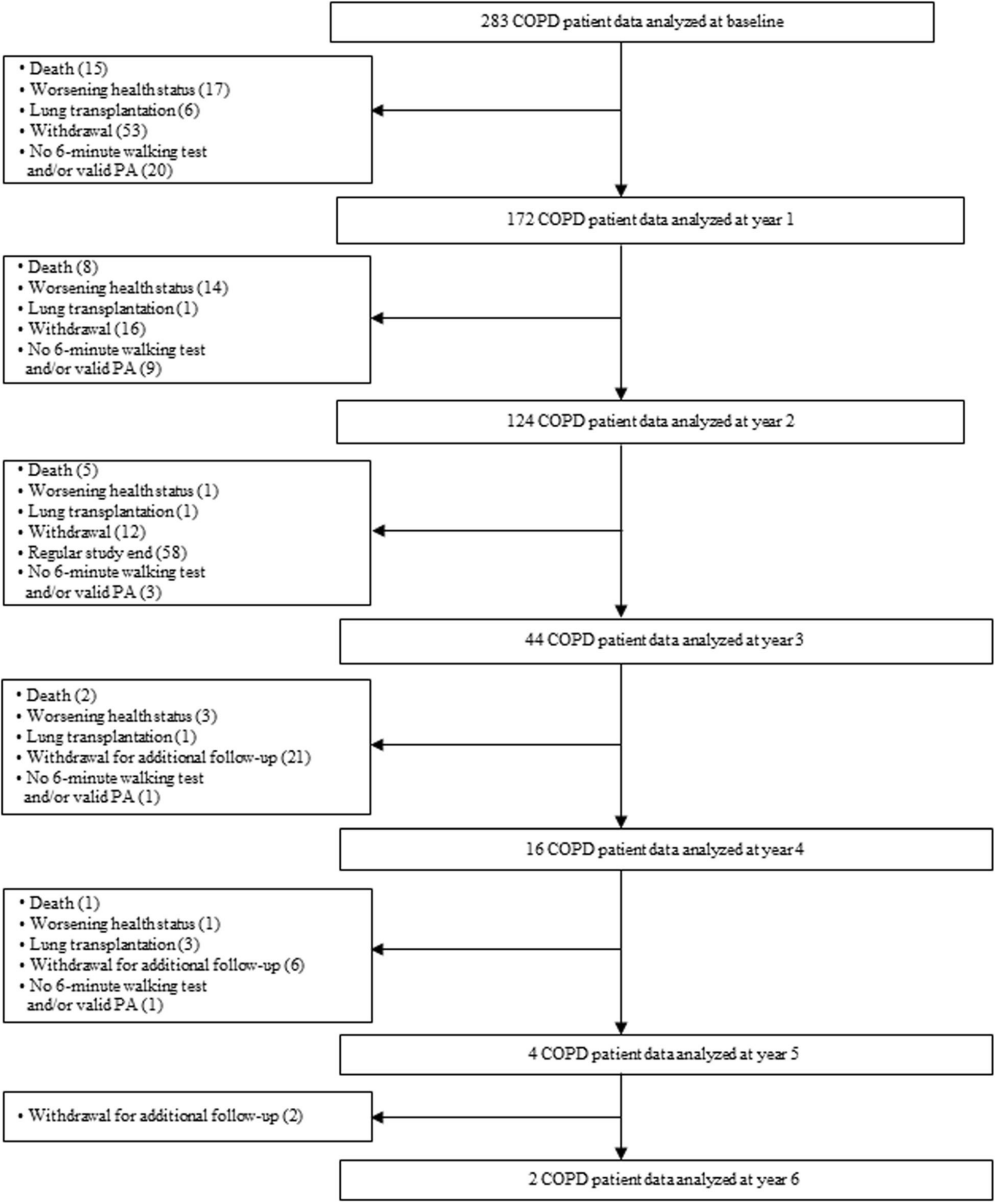
Study flow

Of the 326 patients who agreed to take part, 283 COPD patients (41% spirometric GOLD stage 1/2, 37% stage 3, 23% stage 4) completed baseline visit and were included in the analysis. Patients were asked to participate for at least three study visits, with some patients extended up to 7 study visits. During follow-up, 172 patients were analysed at year 1, 124 at year 2, 44 at year 3, 16 at year 4, 4 at year 5 and 2 at year 6, respectively (Fig. 1). Reasons for missing follow-up visits were withdrawal, not able to participate anymore due to worsening health status, lung transplantation, and death. Mean (min/max) follow-up time was 2.4 (0.5/6.8) years with annual measurements (median (quartiles) time between two visits 1.08 (1.01/1.20)). Comparison between patients who received follow-up and patients who were lost for or failed follow-up showed no differences in baseline PC and PA. The median (quartiles) age was 63 (58/68) years, 65% were male and 24% of the COPD patients were current smokers. Mean (SD) PC was 79.4 (23.1)% of predicted 6MWD corresponding to 418 (125.7) meter. Median (quartiles) PA was 4421 (2522/6863) steps per day. Detailed patient characteristics are presented in Table 1.

**Table 1:** Baseline characteristics

*N* = 283	
Age, y	63 (58/68)
Male / Female, N	184 / 99
BMI, kg/m^2^	25.8 (22.4/28.7)
Waist / Hip ratio	0.97 (0.91/1.03)
Smoker / Former smoker, N (%)	67 (24) / 207 (73)
Pack years, N	40 (30/60)
mMRC score	2 (1/2)
CAT score	15 (11/20)
Anxiety score	4 (2/8)
Depression score	4 (2/7)
*GOLD, N (%)*	
I	25 (9)
II	92 (33)
III	103 (36)
IV	63 (22)
*COPD Risk Group, N (%)*	
A	48 (17)
B	192 (68)
C	9 (3)
D	34 (12)
FEV_1_, % pred.	44 (31/64)
RV/TLC, %	55.6 (11.7)
TLco, ml/mmHg/min	47 (35/68)
PaO_2_, kPa	9.04 (8.21/10.13)
PaCO_2_, kPa	5.03 (4.63/5.45)
SaO_2_, %	94.7 (93.0/96.0)
Number of comorbidities, N	3 (1/4)
Number of exacerbations in the previous year, N	1 (0/1)
Number of severe exacerbations in the previous year, N	0 (0/0)
Frequent exacerbator, N (%)	68 (24)
Survival time, days	2094 (1420/2483)
Retired person, N (%)	191 (68)
6MWD, m	418.3 (125.7)
6MWD, % pred.	79.4 (23.1)
SpO_2_ after 6MWT, %	90 (84/95)
Steps per day, N	4421 (2522/6863)

*BMI* body mass index, *mMRC* modified medical research council, *CAT* COPD assessment test, *FEV*_*1*_ forced expiratory volume in one second, *RV/TLC* residual volume to total lung capacity ratio, *TLco* diffusing capacity of the lung for carbon monoxide, *PaO*_*2*_ partial pressure of oxygen; *PaCO*_*2*_ partial pressure of carbon dioxide; *SaO*_*2*_ oxygen saturation, *6MWD* 6-min walking distance.

### PC-PA quadrants

**Fig. 2:**
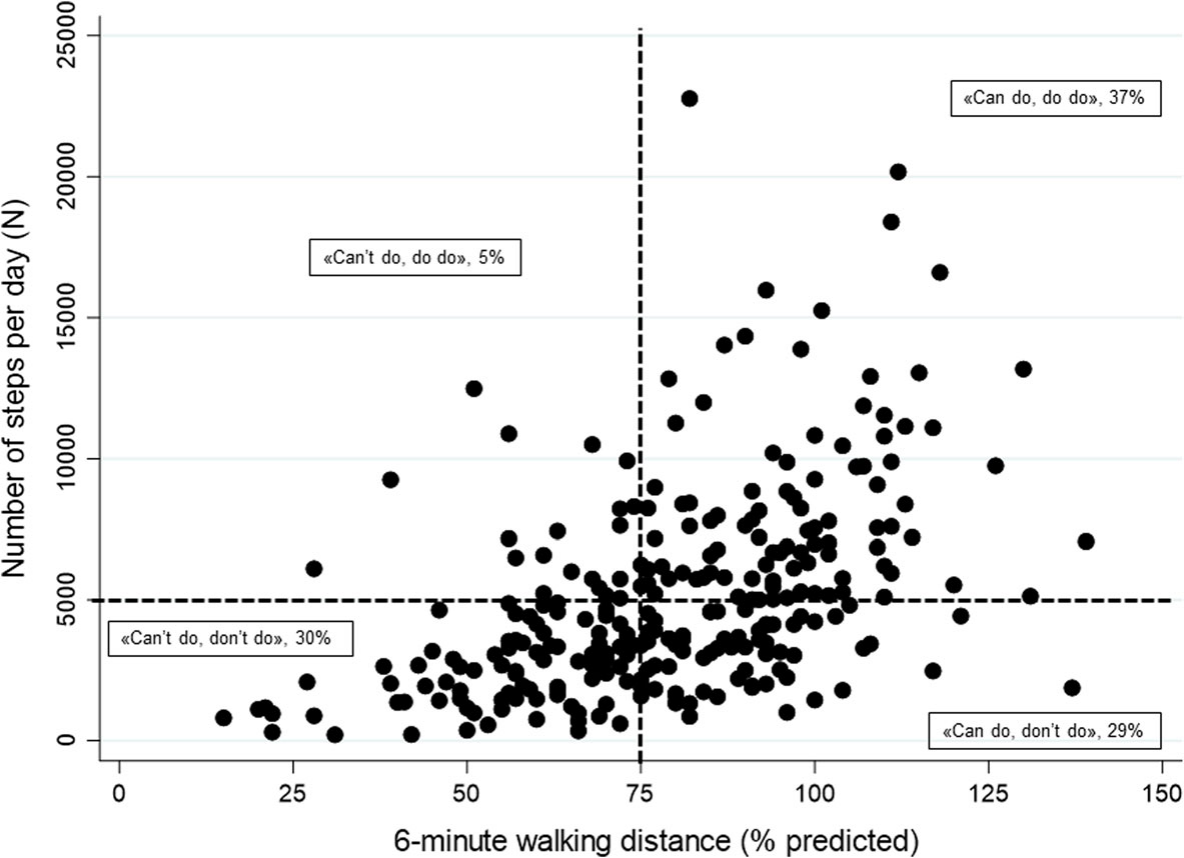
PC-PA quadrant at baseline. 6-min walking distance (x-axis) is potted against number of steps per day (y-axis)

At baseline, 30% (85 patients) were categorized into the “can’t do, don’t do” quadrant, 29% (81 patients) into the “can do, don’t do” quadrant, 5% (13 patients) into the “can’t do, do do” and 37% (104 patients) into the “can do, do do” quadrant (Fig. 2).

### Differences in clinical characteristics in PC-PA quadrants

Comparison of the PC-PA quadrants revealed significant overall differences in various baseline values of clinical characteristics, such as lung function, CAT score and number of comorbidities (Table 2). In year 1, the overall significant differences in clinical characteristics in the PC-PA quadrants partly changed compared to baseline (Additional file 1: Table S1). None of the clinical characteristics showed significant and relevant differences between all of the individual quadrants (Table 3). The PC-PA quadrants could be characterized as follows: I) “can’t do, don’t do” quadrant: most severe and symptomatic COPD patients, several comorbidities II) “can do, don’t do” quadrant: severe but less symptomatic COPD patients, several comorbidities III) “can’t do, do do” quadrant: few patients, severe and symptomatic, less comorbidities IV) “can do, do do” quadrant: mildest and less symptomatic patients, less comorbidities, lowest exacerbation frequency.

**Table 2:** Comparison of clinical characteristics between quadrants at baseline

	“Can’t do, don’t do” *N* = 85	“Can do, don’t do” *N* = 81	“Can’t do, do do” *N* = 13	“Can do, do do” *N* = 104	Overall *p*-value
Age, y	64 (60/69)	64 (58/67)	56 (53/60)*^†^	64 (58/68)^‡^	0.007
Male, N (%)	63 (74)	51 (63)	7 (54)	63 (61)	0.180
BMI, kg/m^2^	24.9 (21.1/28.1)	26.6 (22.8/29.4)	24.0 (22.6/25.8)	26.7 (22.6/29.2)	0.110
Waist/Hip ratio	0.98 (0.90/1.05)	0.98 (0.93/1.02)	0.93 (0.90/1.02)	0.96 (0.89/1.01)	0.250
Smoker, N (%)	11 (13)	25 (31)*	2 (15)	29 (28)*	0.026
mMRC score	3 (2/3)	1 (1/2)*	2 (1/3)	1 (1/2)* ^†‡^	< 0.001
CAT score	18 (14/23)	14 (10/18)*	19 (13/22)	13 (8/19)*	< 0.001
Anxiety score	5 (3/8)	3 (2/6)	7 (5/9)	4.5 (2.0/8.0)	0.080
Depression score	6 (4/8)	4 (2/6)*	5 (2/8)	3.5 (1.0/6.0)*	< 0.001
*GOLD, N (%)*					< 0.001
I	2 (2)	6 (7)	0 (0)	17 (16)*	
II	11 (13)	27 (33)*	6 (46)*	48 (46)*	
III	35 (41)	36 (44)	3 (23)	29 (28)^†^	
IV	37 (44)	12 (15)*	4 (31)	10 (10)*^‡^	
*COPD Risk Group, N (%)*					< 0.001
A	4 (5)	16 (20)*	1 (8)	27 (26)*	
B	68 (80)	54 (67)	10 (77)	60 (58)*	
C	1 (1)	0 (0)	0 (0)	8 (8)*^†^	
D	12 (14)	11 (14)	2 (15)	9 (9)	
FEV_1_, % pred.	31 (25/43)	45 (34/65)*	41 (29/56)	58 (40.5/75.0)*^†^	< 0.001
RV/TLC, %	63 (57/68)	55 (45/65)*	55 (46/62)	52 (43/59)*	< 0.001
TLco, % pred.	36 (28/45)	47 (36/64)*	43 (34/48)	63 (46/78)*^†‡^	< 0.001
PaO_2_, kPa	8.6 (7.6/9.5)	9.0 (8.4/10.1)	9.3 (8.5/9.6)	9.6 (8.8/10.5)*	< 0.001
PaCO_2_, kPa	5.2 (4.7/5.7)	5.0 (4.5/5.3)*	5.0 (4.7/5.2)	4.9 (4.6/5.4)	0.047
SaO_2_, %	93.7 (91.2/95.3)	94.8 (93.3/96.0)	94.8 (94.5/96.0)	95.2 (93.7/96.5)*	0.007
Number of comorbidities, N	3 (2/5)	3 (2/5)	2 (1/2)*^†^	2 (1.0/3.5)*^†^	< 0.001
Exacerbations in the previous year, N	1 (0/2)	0 (0/1)	1 (0/2)	0 (0/1)*^‡^	< 0.001
Severe exacerbations in the previous year, N	0 (0/1)	0 (0/0)*	0 (0/0)	0 (0/0)*	0.006
Frequent exacerbator, N (%)	28 (33)	20 (25)	6 (46)	14 (14)*^‡^	0.004
Survival time, days (*N* = 214)	1550 (836/2268)^a^	2115 (1599/2394)^b^	2390 (1799/3019)^c^	2279 (1944/2839)^d^*^†^	< 0.001
Retired person, N (%)	64 (76)	56 (70)	7 (54)	64 (62)	0.130
6MWD, m	300 (252/349)	450 (390/510)*	360 (300/375)^†^	495 (444/562)*^‡^	< 0.001
6MWD, % pred.	57 (47/65)	87 (77/95)*	61 (56/65)^†^	96 (95/107)*^‡^	< 0.001
SpO_2_ after 6MWT, %	87 (81/92)	90 (85/93)*	91 (87/95)	92 (87/95)*	< 0.001
Steps per day, N	2388 (1329/3357)	3284 (2113/3864)	6590 (6007/9258)*^†^	7553 (5876/9819)*^†^	< 0.001

Values are median (25%/75% quartiles) unless otherwise stated. * *p* < 0.05 vs “can’t do, don’t do”; ^†^*p* < 0.05 vs “can do, don’t do”; ^‡^*p* < 0.005 vs “can’t do, do do”. ^a^*N* = 69; ^b^*N* = 62; ^c^*N* = 8;^d^*N* = 75. *BMI* body mass index, *mMRC* modified medical research council, *CAT* COPD assessment test, *FEV*_*1*_ forced expiratory volume in one second, *RV/TLC* residual volume to total lung capacity ratio, *TLco* diffusing capacity of the lung for carbon monoxide, *PaO*_*2*_ partial pressure of oxygen, *PaCO*_*2*_ partial pressure of carbon dioxide, *SaO*_*2*_ oxygen saturation, *6MWD* 6-min walking distance

**Table 3:** Differences in characteristics between individual quadrants at baseline

	“Can’t do, don’t do” vs “Can do, don’t do”	“Can’t do, don’t do” vs “Can’t do, do do”	“Can’t do, don’t do” vs “Can do, do do”	“Can do, don’t do” vs “Can’t do, do do”	“Can do, don’t do” vs “Can do, do do”	“Can’t do, do do” vs “Can do, do do”
Age, y	−1 (− 3/2)	−8 (− 11/− 4)*	1 (− 3/1)	−7 (− 10/− 4)*	0 (− 2/2)	7 (3/10)*
mMRC score, N	− 1 (− 1/− 1)*	0 (− 1/0)	− 1 (− 2/− 1)*	1 (0/1)	0 (− 1/0)*	− 1 (− 2/0)*
CAT score, N	− 4.3 (−6.1/− 2.4)*	−0.14 (− 4.0/3.7)	−4.6 (− 6.7/− 2.6)*	4.1 (0.6/7.6)	−0.4 (− 2.4/1.6)	−4.5 (− 8.9/− 0.1)
Depression score, N	−2 (− 3/− 1)*	−1 (− 3/2)	− 2 (− 3/− 1)*	1 (−1/4)	0 (− 1/1)	−1 (− 4/1)
FEV_1_, % pred.	13 (8/18)*	7 (−2/20)	23 (17/30)*	−5 (−15/5)	9 (3/16)*	15 (6/25)
RV/TLC, %	−7 (− 10.9/− 4.0)*	−7 (− 14/0)	− 11 (− 14/− 8)*	0 (−7/7)	− 4 (− 8/0)	− 4 (− 11/3)
TLco, % pred.	11 (6/17)*	6 (−2/15)	25 (18/31)*	−5 (− 16/4)	12 (5/18)*	18.5 (8/28)*
Number of comorbidities, N	−1 (− 1/0)	−2 (− 2/− 1)*	− 1 (− 2/− 1)*	−1 (− 2/0)*	−1 (− 1/0)*	1 (0/1)
Exacerbations in the previous year, N	0 (−1/0)	0 (− 1/1)	− 1 (− 1/0)*	1 (0/2)	0 (0/0)	− 1 (− 2/0)*
Severe exacerbations in the previous year, N	0 (0/0)*	0 (0/0)	0 (0/0)*	0 (0/0)	0 (0/0)	0 (0/0)
Survival time, days (N = 214)	332 (49/698)	884 (328/1358)	732 (386/1075)*	461 (− 181/1069)	303 (62/568)*	−84 (− 701/467)
6MWD, m	169.0(182.8/195.2)*	35.2 (−13.2/83.7)	210.7 (185.5/236.0)*	− 133.8 (− 184.4/83.2)*	41.7 (15.6/67.8)	175.5 (123.8/227.2)*
6MWD, % pred.	32 (28/36)*	2 (− 3/8)	40 (36/44)*	−29 (− 36/− 22)*	7 (3/12)	37 (31/44)*
SpO_2_ after 6MWT, %	2 (0/5)*	4 (1/7)	−4 (2/7)*	1 (−1/4)	2 (4/1)	1 (−2/3)
Steps per day, N	692 (263/1108)	4770 (3716/6145)*	5193 (4657/5822)*	3990 (2916/5641)*	4477 (3939/5130)*	349 (− 627/1508)

Values are median (95% CI). *p-value < 0.05. *mMRC* modified medical research council, *CAT* COPD assessment test, *FEV*_*1*_ forced expiratory volume in one second, *RV/TLC* residual volume to total lung capacity ratio, *TLco* diffusing capacity of the lung for carbon monoxide, *6MWD* 6-min walking distance, *SaO*_*2*_ oxygen saturation

Median differences in CAT score between patients with low PC and patients with preserved PC are remarkably above the MID of 2 points, despite lack of statistical significance. Lung function impairment increases from “can do, do do” quadrant to “can do, don’t do”/“can’t do, do do” to “can’t do, don’t do” quadrant, with quite similar values between “can’t do, do do” and “can do, don’t do” quadrants. The median (95% CI) difference in 6MWD was above the MID with 35.2 (− 13.2/83.7) meters in both groups with low PC, without statistical significance. In the two groups with preserved PC, patients in the “can do, don’t do” quadrant showed a relevant but not significant median (95% CI) difference in 6MWD of − 41.7 (− 67.8/− 15.6). Differences in steps per day were rather small between low PA groups and between preserved PA groups, respectively. (Table 3).

### Longitudinal properties of the PC-PA quadrant

After one year, the percentage of patients in the “can’t do, don’t do” quadrant declined from 30 to 19% while the number of patients in the “can do, don’t do” quadrant increased from 29 to 44%. The amount of patients in the remaining two quadrants stayed quite stable (3 and 34%, respectively). Of the 172 patients with at least one follow-up visit, 100 (58%) patients never changed their quadrant affiliation during study period (remainer), while 29 patients (17%) worsened either PC, PA or both (decliner), 20 patients (11%) increased their PC, PA or both (improver), and 24 patients (14%) showed increase and decrease in PC, PA or both (waverer). Follow-up time was significantly longer in the waverer group with median (quartiles) of 3.1 (2.2/4.0) years compared to the remainer (2.1 (1.3/2.5) years) and decliner (2.0 (1.1/3.1) years) (*p* < 0.001 and *p* = 0.011, respectively).

Of the patients in the “can’t do, don’t do” quadrant at baseline, 51% remained in this quadrant while 24% were improver and 24% waverer. In the “can do, don’t do” quadrant, 63% were remainer, 7% decliner, 15% improver and waverer each. 40% of the “can’t do, do do” quadrant remained, while 20% were improver and 40% waverer. The “can do, do do” quadrant at baseline yield 60% remainer, 35% decliner and 5% waverer.

Median yearly changes in most of the clinical characteristics were comparable among the PC-PA quadrants. The significant greater annual decline in number of steps per day in the “can’t do, do do” quadrant compared to the “can do, don’t do” quadrant did not show clinical relevance (median (quartiles) difference of − 449 (− 2728/1284) steps per day) (*p* = 0.001). (Additional file 1: Table S2).

### Differences in clinical characteristics in “changing groups”

None of the clinical parameters at baseline yield meaningful differences between all of the four changing groups (Additional file 1: Table S3). Improver showed a significantly higher CAT score (median (95% CI) difference of 4.6 (1.3/7.9)) compared to remainer, and decliner showed a significantly higher RV/TLC (median (95% CI) difference of 7 (3/10)%) compared to remainer. 6MWD and number of steps per day was lowest in the improver group and highest in the decliner group. Furthermore, median annual changes in clinical characteristics did not differ relevantly between the changing groups despite a relevant median (quartiles) difference in yearly change in number of steps per day between decliners and improvers of − 1120 (− 1722/− 557) steps (*p* < 0.001). (Table 4).

**Table 4:** Comparison of median annual change in clinical characteristics between changing groups

Dependent variable	Remainer *N* = 100	Decliner *N* = 29	Improver *N* = 20	Waverer *N* = 24	*P*-value
Median annual change BMI, kg/m^2^	−0.08 (− 0.75/0.66)	− 0.03 (− 1.09/0.46)	0.01 (− 0.93/1.02)	0 (− 0.71/0.35)	0.740
Median annual change Waist/Hip ratio	0.01 (− 0.03/0.04)	0.01 (− 0.01/0.03)	−0.01 (− 0.03/0.02)	0 (− 0.04/0.02)	0.094
Median annual change mMRC score	0 (0/1)	0 (0/1)	0 (− 1/0)	0 (0/1)	0.210
Median annual change CAT score	1 (−3/3)	2 (−2/4)	−1 (− 5/3)^†^	1 (− 2/3)^‡^	0.042
Median annual change Anxiety score	0 (− 2/1)	0 (− 1/1)	0 (− 2/1)	0 (− 1/2)	0.330
Median annual change Depression score	0 (−2/1)	0 (− 1/2)	− 1 (− 2/0) ^†^	0.5 (− 1.0/1.5)	0.045
Median annual change FEV_1_, % pred.	0 (−4/6)	0 (− 3/5)	3 (− 4/6)	− 2 (− 6/1)*	0.046
Median annual change RV/TLC, %	0 (−5/5)	1 (− 5/5)	0 (− 3.0/2.5)	2.5 (− 2.5/6.0)	0.180
Median annual change TLco, % pred.	−2 (− 6.7/4.0)	− 3 (−9/2)	0 (− 6/6)	− 1.0 (− 7.0/5.0)	0.400
Median annual change PaO_2_, kPa	0.1 (− 0.7/0.6)	−0.21 (− 0.69/0.60)	−0.20 (− 0.82/0.56)	0.08 (− 0.82/0.75)	0.840
Median annual change PaCO_2,_ kPa	0 (− 0.31/0.23)	0.01 (− 0.27/0.20)	−0.10 (− 0.29/0.14)	0.02 (− 0.17/0.42)	0.450
Median annual change SaO_2,_ %	0 (− 1.1/1.2)	0.1 (− 1.5/1.0)	−0.2 (− 1.5/1.0)	0 (− 1/1)	0.860
Median annual change Number of comorbidities	0 (0/0)	0 (0/1)	0 (0/1)	0 (0/0)	0.100
Median annual change Exacerbations in the previous year, N	0 (− 1/0)	0 (− 1/1)	0 (−0.5/0.0)	0 (− 1/1)	0.850
Median annual change Severe exacerbations in the previous year, N	0 (0/0)	0 (0/0)	0 (0/0)	0 (0/0)	0.810
Median annual change 6MWD, m	0 (− 30/30)	−25 (− 60.0/28.7)	11 (− 18/57.5)^†^	0 (−75/35)	0.019
Median annual change 6MWD, % pred.	1.5 (− 3.0/10.0)	0 (−11/7)	9.5 (−2/20)	1 (− 14/14)^‡^	0.005
Median annual change SpO_2_ after 6MWT, %	0 (−3/3)	−1 (− 3/2)	0 (− 3/2)	−1 (− 5/4)	0.540
Median annual change Steps per day, N	− 298 (− 1418/566)	− 1202 (− 2926/− 348)*	− 192 (− 1393/1809)^†^	− 761 (− 1870/665)	< 0.001

Values are median (25%/75% quartiles). *p < 0.05 vs “remainer”; ^†^*p* < 0.05 vs “worsener”; ^‡^*p* < 0.005 vs “improver”

## Discussion

This analysis applyed a recently introduced concept, taking into account the differences in PC and PA for characterizing COPD patients, in our heterogeneous cohort with longitudinal assessments. Patients with low PC and PA presented the worst health status, followed by patients with low PC but preserved PA and patients with preserved PC but low PA. Preserved PC and PA was found in patients with the mildest disease manifestations. Although 42% of the patients changed their quadrant affiliation over time, none of the observed clinical characteristics or their annual changes allowed to distinguish between patients who maintained, improved or decreased physical functioning over time.

Several studies demonstrated the deleterious impact of reduced PC and PA on various outcomes in patients with COPD [28–31]. Despite the effort to clarify the relation of physical functioning and progress of COPD, reasons for the divergent evolution between PC and PA remain unknown. Why the positive effect of enhancements in PC, e.g. with pulmonary rehabilitations, cannot always be transferred into enhanced PA is a matter of current debate and research [32]. Furthermore, it remains to be defined if PA modifying interventions, such as PA counselling, also enhances PC for long term. A previous study by our team revealed that the significant decrease in daily PA over time is not accompanied by a decrease in PC [6]. Consistent with these findings, Koolen et al. [10] developed a new concept in which PC and PA were divided into decreased and preserved. This PC-PA quadrant should enable the identification of physical functioning sub-groups with different clinical characteristics and might be useful in optimizing personalized medicine in COPD patients. Following the call to prove the new PC-PA quadrant concept, we applied the concept to our COPD cohort and investigated if the PC-PA quadrant is applicable for dynamic changes, assessed by longitudinal observations. Comparable to Koolen and colleagues [10], patients in the “can’t do, don’t do” quadrant showed the highest disease burden and patients in the “can do, do do” quadrant the mildest COPD. However, none of the assessed clinical characteristics allowed to distinguish between all of the individual quadrants. Several of the differentiating characteristics found by Koolen et al. [10], such as BMI and sex, could not be confirmed by our study. Moreover, some of the distinguishing characteristics found to be significant in the baseline assessment were not maintained significant in the following year. The largest mismatch was found in the “can do, don’t do” patients. Koolen et al. [10] reported the smallest prevalence in these patients and described them as “lazy”, with the highest BMI and low exacerbation history. Our data suggested that these patients show a comparable severity of COPD with the “can’t do, do do” patients, but with slightly lower symptoms and a higher PC. We thus hypothesize that the already severe respiratory impairment did not translate into a low PC yet, but will lead to an impairment in PC with a delay in time. This finding could also deliver an explanation why the annual decrease in PA is not accompanied by a decrease in PC [6], leading to the assumption that PC may decrease with delay while PA impairment goes in line with disease worsening. To address this, longitudinal studies are needed in which early disease stages are monitored and the onset of PA impairment will be compared with the onset of PC impairment. Furthermore, this raises the question if regular assessment of PA in clinical practice would be more sensitive to detect progressive deterioration of COPD compared to the commonly used PC. The PC-PA quadrant concept was not able to project the dynamic course of the disease. Almost half of the patients changed their PC-PA quadrant over time but changes in quadrant affiliation were not connected to clinical characteristics or their annual changes. We therefore assume that detailed characterization of the four PC-PA quadrants would not be adequate. However, further studies are needed to confirm whether patients in the “can do, don’t do” quadrant show severe disease burden and if these patients would be more responsive for PA enhancement after pulmonary rehabilitations.

The current study has some limitations. Patients with pulmonary rehabilitation within 3 months prior to the baseline evaluation were not included at this time point. However, we cannot exclude that a small number of severe patients underwent pulmonary rehabilitation during the study period, potentially increased the group of waverers. Furthermore, the number of patients in the “can’t do, do do” quadrant is quite small but seems to represent the real-world distribution of COPD patients.

## Conclusion

In conclusion, we found no clinical characteristics to distinguish significantly and clinically meaningful between the PC-PA quadrants. Furthermore, the PC-PA quadrant concept seems not to be able to reflect disease process in COPD patients with a follow-up up to six years. However, the already low PA and preserved PC in the “can do, don’t do” quadrant raises the question if regularly assessment of PA in clinical practice would be more sensitive to detect progressive deterioration of COPD compared to the commonly used PC.

## Supplementary information

**Additional file 1: Table S1.** Comparison of clinical characteristics between quadrants at year 1. **Table S2.** Comparison of change in clinical characteristics between quadrants. **Table S3.** Comparison of clinical characteristics at baseline between changing groups.

## Data Availability

The datasets used and analysed during the current study are available from the corresponding author on reasonable request.

## Abbreviations

6MWD: 6-min walking distance
6MWT: 6-min walking test
AE: Acute exacerbation
ATS: American thoracic society
BMI: Body mass index
CAT: COPD Assessment Test
CI: Confidence interval
COPD: Chronic obstructive pulmonary disease
FEV_1_: Forced expiratory volume in 1 s
FVC: Forced vital capacity
GOLD: Global initiative for chronic obstructive lung disease
HADS: Hospital Anxiety and Depression Scale
MID: Minimal important difference
mMRC: Modified medical research council
PA: Physical activity
PaCO2: Partial pressure of carbon dioxide
PaO2: Partial pressure of oxygen
PC: Physical capacity
SaO2: Oxygen saturation
TOPDOCS: The obstructive pulmonary disease outcomes cohort of Switzerland
