# Supplementary material for: “Can do, don’t do” are not the lazy ones: a longitudinal study on physical functioning in patients with COPD
Source: Respir Res. 2020 Jan 20;21:27. doi: 10.1186/s12931-020-1290-9 (PMC6972031; doi:10.1186/s12931-020-1290-9)
Supplement: Supplementary file 1 — Additional file 1: Table S1. Comparison of clinical characteristics between quadrants at year 1. Table S2. Comparison of change in clinical characteristics between quadrants. Table S3. Comparison of clinical characteristics at baseline between changing groups. [file 12931_2020_1290_MOESM1_ESM.docx]

**“Can do, don’t do” are not the lazy ones: a longitudinal study on physical functioning in patients with COPD**

**Additional file**

Noriane A Sievi^1^ MSc, Thomas Brack MD^2^, Martin H Brutsche MD^3^, Martin Frey MD^4^, Sarosh Irani MD^5^, Jörg D Leuppi MD^6^, Robert Thurnheer MD^7^, Malcolm Kohler MD^1,8,^ and Christian F Clarenbach MD^1^

^1^Pulmonary Division, University Hospital Zurich, Switzerland

^2^Pulmonary Division, Cantonal Hospital of Glarus, Switzerland

^3^Pulmonary Division, Cantonal Hospital of St. Gallen, Switzerland

^4^Pulmonary Division, Clinic Barmelweid, Switzerland

^5^Pulmonary Division, Cantonal Hospital of Aarau, Switzerland

^6^University Department of Medicine, Cantonal Hospital Baselland and University of Basel, Switzerland

^7^Pulmonary Division, Cantonal Hospital of Münsterlingen, Switzerland

^8^Zurich Centre for Integrative Human Physiology, University of Zurich, Switzerland

| **Table S1. Comparison of clinical characteristics between quadrants at year 1.** | | | | | |
| --- | --- | --- | --- | --- | --- |
|  | **“Can’t do, don’t do”**  **N=32** | **“Can do, don’t do”**  **N=76** | **“Can’t do, do do”**  **N=5** | **“Can do, do do”**  **N=59** | **Overall p-value** |
| Age, y | 69 (63/72) | 66 (64/70) | 61 (56/64) | 63 (57/69)*^†^ | 0.007 |
| Male, N (%) | 19 (59) | 51 (67) | 4 (80) | 38 (64) | 0.780 |
| BMI, kg/m^2^ | 24.9 (21.6/28.1) | 25.5 (21.4/29.5) | 19.6 (18.6/22.6) | 25.2 (23.0/28.9) | 0.074 |
| Waist/Hip ratio | 0.96 (0.91/1.04) | 0.99 (0.94/1.03) | 0.91 (0.91/0.91) | 0.97 (0.91/1.03) | 0.230 |
| Smoker, N (%) | 4 (13) | 15 (20) | 3 (60) | 17 (29) | 0.057 |
| mMRC score | 3 (2/3) | 2 (1/2)* | 2 (2/2) | 1 (0/1)*^†‡^ | <0.001 |
| CAT score | 21 (17/25) | 14 (9/19)* | 18 (11/27) | 10 (6/15)* | <0.001 |
| Anxiety score | 5.5 (2/8) | 3 (2/5) | 6 (4/7) | 4 (2/6) | 0.063 |
| Depression score | 5 (2/10) | 3 (2/5) | 5 (4/6) | 3 (1/6)* | 0.020 |
| *GOLD, N (%)* |  |  |  |  | <0.001 |
| I | 2 (6) | 8 (11) | 0 (0) | 17 (29)*^†^ |  |
| II | 2 (6) | 24 (32)* | 2 (40)* | 29 (49)*^†^ |  |
| III | 12 (38) | 34 (45) | 2 (40) | 10 (17)*^†^ |  |
| IV | 16 (50) | 10 (13)* | 1 (20) | 3 (5)* |  |
| *COPD Risk Group, N (%)* |  |  |  |  | 0.003 |
| A | 0 (0) | 15 (20)* | 1 (20)* | 24 (41)*^†^ |  |
| B | 29 (91) | 49 (64)* | 4 (80) | 29 (49)* |  |
| C | 0 (0) | 5 (7) | 0 (0) | 3 (5) |  |
| D | 3 (9) | 7 (9) | 0 (0) | 3 (5) |  |
| FEV_1_, % pred. | 31 (24.0/39.5) | 46 (34.0/57.0)* | 44 (40.0/56.0) | 68 (51.0/82.0)*^†^ | <0.001 |
| RV/TLC, % | 64 (59/70) | 58 (49/62)* | 60 (59/60) | 48.5 (41.0/57.0)*^†‡^ | <0.001 |
| TLco, % pred. | 37 (25/41) | 42 (34/55)* | 36 (29/41) | 64 (47/76)*^†‡^ | <0.001 |
| PaO_2_, kPa | 8.7 (7.8/9.5) | 9.3 (8.0/10.0) | 9.8 (9.3/9.8) | 9.1 (8.7/10.3) | 0.340 |
| PaCO_2_, kPa | 5.1 (4.6/5.7) | 4.8 (4.5/5.3) | 4.7 (4.2/4.9) | 4.8 (4.5/5.1) | 0.090 |
| SaO_2_, % | 94 (91.6/95.5) | 95 (92.4/96.0) | 96 (95.2/96.0) | 95 (93.4/96.0) | 0.190 |
| Number of Comorbidities, N | 4 (2/5) | 3 (2/5) | 2 (1/2)*^†^ | 2 (1/4)*^†^ | 0.003 |
| Exacerbations in the previous year, N | 1 (0/2) | 0 (0/1)* | 1 (0/1) | 0 (0/1)* | <0.001 |
| Severe exacerbations in the previous year, N | 0 (0/1) | 0 (0/0) | 0 (0/0) | 0 (0/0)* | 0.002 |
| Frequent exacerbator, N (%) | 10 (31) | 9 (12)* | 1 (20) | 4 (7)* | 0.013 |
| Survival time, days (N=133) | 1899 (1049/2195)^a^ | 2268 (1845/2570)^b^* | 2560 (2098/2629)^c^ | 2285 (2038/2666)^d^* | 0.002 |
| Retired person, N (%) | 28 (90) | 63 (85) | 4 (80) | 34 (59)* ^†‡^ | 0.001 |
| 6MWD, m | 245 (170/293) | 450 (375/505)* | 330 (300/371) | 540 (450/584)*^†‡^ | <0.001 |
| 6MWD, % pred. | 48.3 (17.0) | 95.8 (18.3)* | 59.4 (9.6)^†^ | 109.5 (20.0)*^†‡^ | <0.001 |
| SpO_2_ after 6MWT, % | 86 (79/92) | 91 (86/94) | 91 (87/93) | 94 (87/97)* | 0.002 |
| Steps per day, N | 1084 (437/3308) | 2640 (2173/3976)* | 5664 (5443/9317)*^†^ | 7651 (6312/10589)*^†^ | <0.001 |
| Values are median (25%/75% quartiles) unless otherwise stated. * p<0.05 vs “can’t do, don’t do”; ^†^ p<0.05 vs “can do, don’t do”; ^‡^ p<0.005 vs “can’t do, do do”. ^a^N=27; ^b^N=60; ^c^N=3;^d^N=44. BMI: body mass index; mMRC: modified medical research council; CAT: COPD assessment test; FEV_1_: forced expiratory volume in one second; RV/TLC: residual volume to total lung capacity ratio; TLco: diffusing capacity of the lung for carbon monoxide; PaO_2_: partial pressure of oxygen; PaCO_2_: partial pressure of carbon dioxide; SaO_2_: oxygen saturation; 6MWD: 6-minute walking distance. | | | | | |

| **Table S2. Comparison of change in clinical characteristics between quadrants.** | | | | | |
| --- | --- | --- | --- | --- | --- |
|  | **Can’t do, don’t do** | **Can do, don’t do** | **Can’t do, do do** | **Can do, do do** | **P-value** |
| Median annual change BMI, kg/m^2^ | -0.18 (-1.04/0.58) | -0.21 (-0.93/0.45) | 0 (-0.13/1.07) | 0 (-0.64/0.68) | 0.180 |
| Median annual change Waist/Hip ratio | 0 (-0.04/0.04) | 0 (-0.03/0.04) | -0.01 (-0.03/0.01) | 0.01 (-0.03/0.04) | 0.740 |
| Median annual change mMRC score | 0 (0/1) | 0 (0/1) | 0 (0.0/0.5) | 0 (0/0) | 0.450 |
| Median annual change CAT score | 0 (-3/4) | 1 (-2.5/3.0) | 1 (-1.0/2.5) | 1 (-3/3) | 0.910 |
| Median annual change Anxiety score | 0 (-2/1) | 1 (-1/2) | 0 (-1/1) | 0 (-2/1) | 0.033 |
| Median annual change Depression score | 0 (-2/2) | 0 (-1/1) | 1 (-1/1) | 0 (-2/1) | 0.520 |
| Median annual change FEV_1_, % pred. | 0 (-4/3) | 0 (-4/6) | 0 (-6/3) | 0 (-5/6) | 0.720 |
| Median annual change RV/TLC, % | 0 (-3/7) | 1 (-4/5) | 1 (-5/8) | 0 (-5/5) | 0.890 |
| Median annual change TLco, % pred. | -2 (-8/3) | -1.0 (-6.8/7.0) | -4 (-7/5) | -2 (-7.0/2.5) | 0.270 |
| Median annual change PaO_2_, kPa | 0.13 (-0.79/0.90) | -0.02 (-0.67/0.62) | -0.37 (-1.05/0.50) | 0 (-0.69/0.58) | 0.590 |
| Median annual change PaCO_2_, kPa | 0.03 (-0.35/0.31) | 0.01 (-0.21/0.30) | 0.03 (-0.10/0.20) | 0 (-0.31/0.20) | 0.600 |
| Median annual change SaO_2_, % | 0.40 (-1.85/2.00) | 0 (-1/1) | -0.25 (-2.1/0.35) | 0 (-1/1) | 0.530 |
| Median annual change Number of comorbidities, N | 0 (0/1) | 0 (0/0) | 0 (0/0) | 0 (0/0) | 0.057 |
| Median annual change Exacerbations in the previous year, N | 0 (-1/1) | 0 (-1/0) | 0 (0/0) | 0 (0/0) | 0.950 |
| Median annual change Severe exacerbations in the previous year, N | 0 (0/0) | 0 (0/0) | 0 (0/0) | 0 (0/0) | 0.330 |
| Median annual change 6MWD, m | 0 (-37/29) | 0 (-45/25) | 17 (0.0/57.5) | 0 (-42.9/36.0) | 0.087 |
| Median annual change 6MWD, % pred. | 1.5 (-6.5/14.5) | 1 (-6/10) | 6 (1/17) | 2 (-5/15) | 0.150 |
| Median annual change SpO_2_ after 6MWT, % | 1 (-3/4) | 0 (-4/3) | -0.5 (-4.0/2.5) | 0 (-2/2) | 0.530 |
| Median annual change Steps per day, N | -298 (-778/0) | -273 (-1023/580)* | -1327 (-2860/261)^†^ | -1064 (-2931/756) | 0.002 |
| Values are median (25%/75% quartiles). *p<0.05 vs “remainer”; ^†^p<0.05 vs “worsener”; ^‡^p<0.005 vs “improver”. | | | | | |

| **Table S3. Comparison of clinical characteristics at baseline between changing groups.** | | | | | |
| --- | --- | --- | --- | --- | --- |
|  | **Remainer**  **N=100** | **Decliner**  **N=29** | **Improver**  **N=19** | **Waverer**  **N=24** | **P-value** |
| Age, y | 64 (60/69) | 65 (62/69) | 62 (56/69) | 63 (58.0/67.5) | 0.530 |
| Male, N (%) | 69 (69) | 15 (52) | 15 (79) | 13 (54) | 0.120 |
| BMI, kg/m^2^ | 25.9 (23.0/28.4) | 24.0 (22.5/27.6) | 27.6 (22.6/30.4) | 24.4 (20.2/27.6) | 0.120 |
| Waist/Hip ratio | 0.97 (0.92/1.02) | 0.95 (0.90/1.01) | 1.01 (0.92/1.09) | 0.93 (0.87/1.01) | 0.080 |
| Smoker, N (%) | 28 (28) | 5 (17) | 5 (26) | 4 (17) | 0.510 |
| mMRC score | 1 (12) | 2 (1/2) | 2 (1/2) | 2 (1/2) | 0.270 |
| CAT score | 14 (9/19) | 15 (9/20) | 17 (15/23)* | 14 (11.0/18.5) | 0.047 |
| Anxiety score | 3 (2/7) | 3.5 (0.5/7.0) | 7 (4/9) | 4 (2/8) | 0.130 |
| Depression score | 4 (2/6) | 3 (2/5) | 7 (5/7) | 4 (2/6) | 0.053 |
| *GOLD, N (%)* |  |  |  |  | 0.360 |
| I | 14 (14) | 0 (0) | 2 (11) | 2 (8) |  |
| II | 34 (34) | 10 (35) | 8 (42) | 5 (21) |  |
| III | 36 (36) | 15 (52) | 6 (32) | 10 (42) |  |
| IV | 16 (16) | 4 (14) | 3 (16) | 7 (29) |  |
| *COPD Risk Group, N (%)* |  |  |  |  | 0.490 |
| A | 22 (22) | 7 (24) | 1 (5) | 2 (8) |  |
| B | 65 (65) | 17 (59) | 14 (74) | 18 (75) |  |
| C | 4 (4) | 2 (7) | 0 (0) | 1 (4) |  |
| D | 9 (9) | 3 (10) | 4 (21) | 3 (13) |  |
| FEV_1_, % pred. | 48.5 (34.0/68.5) | 41 (33/53) | 56 (34/71) | 36.5 (29/58) | 0.068 |
| RV/TLC, % | 53 (44/61) | 60.4 (52.8/67.0)* | 53 (45/63) | 56.5 51.8/64.5) | 0.015 |
| TLco, % pred. | 54.5 (36.0/71.0) | 47.0 (38.0/61.5) | 50 (35/71) | 41 (35/52) | 0.130 |
| PaO_2_, kPa | 9.2 (8.7/10.1) | 9.6 (7.9/10.4) | 9.1 (8.4/10.4) | 9.1 (8.1/10.3) | 0.970 |
| PaCO_2_, kPa | 4.9 (4.5/5.3) | 5.0 (4.8/5.5) | 5.1 (4.7/5.4) | 4.8 (4.4/5.3) | 0.240 |
| SaO_2_, % | 95 (93.4/96.0) | 95.2 (90.7/96.0) | 94.8 (94.0/96.1) | 94.8 (93.0/96.0) | 0.970 |
| LVRS during study, N (%) | 4 (4) | 4 (14) | 2 (11) | 3 (13) | 0.210 |
| Number of comorbidities, N | 3 (1/4) | 2 (1/4) | 3 (2/4) | 3 (2/4) | 0.710 |
| Number of exacerbations in the previous year, N | 0 (0/1) | 1 (0/2) | 1 (0/2) | 1 (0/2) | 0.470 |
| Number of severe exacerbations in the previous year, N | 0 (0/0) | 0 (0/1) | 0 (0/0) | 0 (0/0) | 0.800 |
| Frequent exacerbator, N (%) | 19 (19) | 9 (31) | 5 (26) | 8 (33) | 0.340 |
| Survival time, days (N=133) | 2172 (1859/2456)^a^ | 2239 (189572410)^b^ | 2268 (2132/2595)^c^ | 2445 (1766/2677)^d^ | 0.370 |
| Retired person, N (%) | 74 (74) | 23 (79) | 11 (65) | 15 (63) | 0.480 |
| 6MWD, m | 451 (345/526) | 450 (375/495) | 390 (360/450) | 378 (361/473) | 0.270 |
| 6MWD, % pred. | 86 (72/102) | 91 (77/97) | 70 (66/90) | 72 (69/90) | 0.031 |
| SpO_2_ after 6MWT, % | 91 (85/95) | 88 (81/94) | 91.5 (89/95) | 90 (86/94) | 0.600 |
| Steps per day, N | 4504 (2028/8077) | 5766 (5161/7550) | 3409 (2640/4456)^†^ | 3589 (2561/5751) | 0.022 |
| *Quadrant at BL* |  |  |  |  | <0.001 |
| Can’t do, don’t do, N (%) | 21 (21) | 0 (0)* | 10 (53)*^†^ | 10 (42)*^†^ |  |
| Can do, don’t do, N (%) | 34 (34) | 4 (14)* | 8 (42) ^†^ | 8 (33) |  |
| Can’t do, do do, N (%) | 2 (2) | 0 (0) | 1 (5) | 2 (8) |  |
| Can do, do do, N (%) | 43 (43) | 25 (86)* | 0 (0)*^†^ | 4 (17)*^†^ |  |
| Values are median (25%/75% quartiles) unless otherwise stated. *p<0.05 vs “remainer”; ^†^p<0.05 vs “worsener”; ^‡^p<0.005 vs “improver”. ^a^N=82; ^b^N=20; ^c^N=11;^d^N=20 | | | | | |
